# Psychogenic nonepileptic seizures: facts, emotions, and money

**DOI:** 10.1055/s-0042-1760107

**Published:** 2022-12-28

**Authors:** Luciano de Paola

**Affiliations:** 1 Universidade Federal do Paraná, Hospital de Clínicas, Serviço de Epilepsia e Eletrencefalografia, Curitiba PR, Brazil.

An excellent review by Aybek and Perez was recently published on the British Medical Journal, discussing various aspects on functional neurological disorders (FNDs). 1 In their view, FNDs, previously a diagnosis of exclusion, have evolved and is now a rule-in diagnosis, with available treatments. That, in itself, represents a true change in paradigm, when approaching FNDs cases. By the same token (and just a few months later), Hallet and coworkers discussed the so called, new subtypes of FNDs. These, include functional seizures, functional movement disorders, persistent perceptual postural dizziness, and functional cognitive disorder, all sharing, among different features, overactivity of the limbic system and a dysfunctional brain network responsible for giving movement the sense of voluntariness. 2

Psychogenic nonepileptic seizures (PNES), undoubtedly, play a major role in this set of conditions that not rarely makes neurologists uncomfortable on both, diagnosis and management approaches. Insecurity comes from facing a paroxysmal event that resembles an epileptic seizure (ES), often a dramatic diagnosis and justly regarded as a neurological emergency. However, in the case PNES, without the neurophysiological underpinnings of epilepsy (i.e., no concomitant changes on the electroencephalogram). During their training, neurologists become far more familiar with ES protocols, usually based on an established sequence of antiseizure medications (ASM), making it “conveniently easier” to embrace a “seizure disorder” diagnosis, rather than challenge the “inhospitable territory”, represented by the frontier between Neurology and Psychiatry, the homeland of PNES.

Asadi-Pooya, the current leading author on PNES, provided a concise review, 3 in which 3 messages are pivotal: (a) PNES are very common, representing 5-10% of outpatients in epilepsy clinics and 20-40% of inpatients in epilepsy monitoring units, with an overall prevalence of 2-33 cases per 100,000 people. Fact: general neurologists will see PNES cases in their routine practice; (b) up to 20-30% of adults carrying the diagnosis of epilepsy might be actually misdiagnosed. Fact: neurologists have been wrong, either at the time of initial diagnosis or perpetuating the error by simply repeating ASM prescriptions; and (c) neurobiology of PNES is poorly understood and neurologists have a tendency to refer these patients to mental health professionals and abandon the cases, whereas it would be desirable to follow them and provide support for ASM tapper, prevent inappropriate treatment and effectively treat comorbid neurological conditions. Fact: neurologists frequently feel scared or annoyed by PNES. Regretfully, this kind of effect represents more likely the rule than the exception and provide fertile soil for continuing mistakes and stigma. This is also a broad and cross-cultural phenomenon.

Almost 20 years ago, along with a number of parallel sessions held at the 25 ^th^ International Epilepsy Congress (Lisbon, Portugal, October 12-16,2003), one particular panel called attention. That morning, a crowded and curious audience focused on “Multicultural Aspects of Nonepileptic Seizures” 4 . Case series from studies performed in the USA, Brazil, Taiwan, Lebanon and India were thoroughly presented and compared. Both, their commonalities and unique expressions, led to an enthusiastic round of discussion, marking the start of a new and promising line of research. Although definitions, clinical phenomena, diagnostic tools and even therapeutic approach tend to be similar, social and environmental aspects differ amongst different cultures, motivating a swarm of publications that translate local experiences on various aspects of FND, including PNES. Dorche *et al* reported on this noticeable risen in the international research on PNES, particularly in the past two decades. 5 Over 1000 papers were published, roughly 80% coming from the US and different European countries, followed by Asia, Oceania, South America and Africa. Their conclusion: in spite of growing interest, there is still great disparity regarding research on PNES, calling for a global campaign in order to inform and educate the World on this issue.

This is why the efforts of the Israeli group, led by Saker *,* along with their colleagues from Turkey, with Pekos as leading author, are very welcome to the current issue of Arquivos de Neuropsiquiatria. These two papers translated the reality of care to PNES patients at their settings, thus providing basis for comparison with other institutions, over different regions of the World. At the same time, their studies enlightened ER staff personnel and general neurologists on the forthcoming issues, whenever seeing their next PNES case. The authors addressed, respectively, the emotional attitudes of health care providers and the economic burden of PNES.

As it seems, the diagnosis of PNES itself could be technically and easily established by most of the 47 professionals who answered the questionnaire, proposed by Saker and colleagues. 6 This is the glass half full. The flip side of the coin is the same professionals perceived feelings of “anger” and “time wasting” whenever caring for PNES patients. Certainly, the responders' thoughts and feelings reflect many health care providers' opinions on PNES patients. In fact, a systematic review gathering the views of at least 3900 health care practitioners, indicated the following points: (a) uncertainty about diagnosis and treatment of PNES; (b) the perception of PNES as associated with psychological factors; (c) that PNES patients are felt as challenging and frustrating cases; (d) mixed views about who is responsible for PNES patientś care and, (e) PNES diagnosis associated with lesser severity when compared to epilepsy and greater degree of volition involved in the events. 7 Their conclusions simultaneously show the broadness of the matter and mark the targets for education.

Pekos and coworkers concluded that early diagnosis and psychosocial support are key to reduce the financial burden on the health system, as well as, to increase the quality of life of PNES patients, after analyzing charts on 134 patients who applied to receive disability reports. 8 This is certainly a wishful goal and, at the same time, a rather challenging one. An editorial at Neurology discussed the delay between the onset of seizures and the correct diagnosis of PNES, estimating an average of 7 years, along with other shocking figures. The same authors estimated a cost of $100 to $900 million per year on repeated workups and treatments for what is wrongly diagnosed as epilepsy. 9 Sadly, exactly 10 years later, Salinsky et al reviewed records on 28 Veterans diagnosed with PNES and concluded that health care utilization did not improve during the 3 years following the diagnosis of PNES, compared to the 3 years prior to the diagnosis. 10 Clearly, a lot of work ahead of us in this field. And again, evidence that misdiagnosis and the resulting cost when it comes to PNES represent an international health care issue.

Thus, PNES remains a challenging area in the universe of FND, a long trajectory from sorcery and hysteria to specific neuronal circuitries disclosed by functional neuroimaging. A journey far from over on an increasingly exciting path.
